# Endoscopically Injectable Shear‐Thinning Hydrogels Facilitating Polyp Removal

**DOI:** 10.1002/advs.201901041

**Published:** 2019-07-30

**Authors:** Yan Pang, Jinyao Liu, Zaina L. Moussa, Joy E. Collins, Shane McDonnell, Alison M. Hayward, Kunal Jajoo, Robert Langer, Giovanni Traverso

**Affiliations:** ^1^ Department of Ophthalmology Ninth People's Hospital Shanghai Key Laboratory of Orbital Diseases and Ocular Oncology Shanghai Jiao Tong University School of Medicine Shanghai 200011 China; ^2^ Department of Chemical Engineering and Koch Institute for Integrative Cancer Research Massachusetts Institute of Technology Cambridge MA 02139 USA; ^3^ Institute of Molecular Medicine State Key Laboratory of Oncogenes and Related Genes Shanghai Institute of Cancer Renji Hospital Shanghai Jiao Tong University School of Medicine Shanghai 200127 China; ^4^ Department of Chemical Engineering and Koch Institute for Integrative Cancer Research Division of Comparative Medicine Massachusetts Institute of Technology Cambridge MA 02139 USA; ^5^ Division of Gastroenterology Brigham and Women's Hospital Harvard Medical School Boston MA 02115 USA; ^6^ Department of Chemical Engineering and Koch Institute for Integrative Cancer Research Harvard‐MIT Division of Health Sciences and Technology Massachusetts Institute of Technology Cambridge MA 02139 USA; ^7^ Department of Mechanical Engineering Massachusetts Institute of Technology Cambridge MA 02139 USA

**Keywords:** endoscopically injectable, removal of polyps, shear‐thinning hydrogels, submucosal

## Abstract

Submucosal elevation, the process of instilling material in the submucosal space for separation of the surface mucosa and deeper muscularis layer, is a significant aspect of the endoscopic mucosal resection of large lesions performed to facilitate lesion removal and maximize safety. Submucosal injection, when applied, has historically been performed with normal saline, though this is limited by its rapid dissipation; solutions ideally need to be easily injectable, biocompatible, and provide a long‐lasting submucosal cushion with a desirable height. Here, reported is a new set of materials, endoscopically injectable shear‐thinning hydrogels, meeting these requirements because of their biocompatible components and ability to form a solid hydrogel upon injection. These findings are supported by evaluation in a large animal model and ultimately demonstrate the potential of these shear‐thinning hydrogels to serve as efficient submucosal injection fluids for cushion development. Given these unique characteristics, their broad application in mucosal resection techniques is anticipated.

## 1 Introduction

Polypectomy remains the single intervention facilitating the interruption of polyp progression toward cancer. Approximately over 15 million colonoscopies are performed annually in the United States with ≈20–25% of these involving polypectomies many of which are performed with the aid of a submucosal injection to facilitate resection^1^, ^2^, ^3^ Endoscopic mucosal resection is a commonly used minimally invasive technique applied in the removal of large polyps (≥2 cm) and early stage tumors because of its simplicity and safety.^4^, ^5^ This is often assisted through an initial submucosal injection used for the establishment of a cushion between the surface mucosa and muscular tissue layers. Since its description in 1984,^6^ normal saline (0.9 wt% sodium chloride) has been the main injection fluid used for endoscopic mucosal resection. Recently, other fluids including hypertonic saline, hypertonic dextrose water, autologous blood, sodium hyaluronate, glycerol, hyaluronic acid, succinylated gelatin, hydroxypropyl methylcellulose, poloxamer, and fibrinogen have been applied to prolong cushion stability by increasing the viscosity of the fluid.^7^, ^8^, ^9^, ^10^, ^11^, ^12^, ^13^ However, the application of these solutions has been largely restricted by unmet safety profiles and durations. Specifically, the heights of cushions elevated by hypertonic saline, dextrose water, and glycerol reduce to less than 50% in 30 min.^14^, ^15^, ^16^ Additionally, injection solutions showing significantly prolonged duration can be associated with administration challenges. For example, carboxymethylcellulose solutions can require a special 18 gauge submucosal injection needle catheter to minimize injection resistance because of its high viscosity.^17^, ^18^ Moreover, hyaluronic acid potentially stimulates the growth of residual tumor tissues.^19^ Fibrinogen and autologous blood are biologic materials which may increase the risk of infection via contamination.^20^, ^21^ Submucosal injection solutions play a critical role for the successful, safe, and intact removal of lesions as they not only lift up diseased mucosa but also provide a gap between the mucosal and deeper layer of tissues facilitating the resection of lesions. Ensuring complete, safe resection stands to mitigate the risk of local recurrence.^22^ Hence, an ideal injection solution for submucosal elevation needs to be biocompatible, easily injectable, and provide durable submucosal cushions.

One potential set of materials with enhanced biocompatibility and prolonged duration of lift is hydrogels due to their high‐water content and stiffness, which can simultaneously minimize toxicity and resist diffusion.^23^ Unfortunately, conventional hydrogels crosslinked by chemical bonds or physical interactions are generally not amenable to injection through an endoscopic needle.^24^ In situ formation of hydrogels is an effective approach to solve this problem and has been widely used to facilitate in vivo application of hydrogels. However, the formation of hydrogels by in situ chemical reactions requires the injection of two or more components simultaneously,^25^ which is challenging for endoscopic submucosal injection. Additionally, the physical crosslinking of hydrogels such as thermo‐triggered gelation has risk of obstructing the endoscopic needle during injection.^26^, ^27^ Here, we describe the development of endoscopically injectable shear‐thinning hydrogels (EISHs) that can be easily injected through the endoscopic needle and immediately recover their mechanical properties as a solid gel upon deployment in the submucosal compartment. These gels demonstrate their potential to serve as safe and easily injectable agents that can provide durable submucosal cushions in a large animal model. Given their unique characteristics, we believe EISHs could prove useful in endoscopic mucosal resection technique for accurate removal of polyps and early stage tumors.

## 2 Results and Discussion

### 2.1 Design and Preparation of EISHs

**Figure 1:**
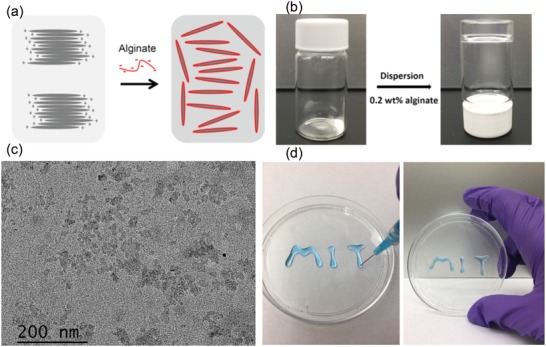
EISH platform and preparation approach. a) Schematic illustration of exfoliated clay nanosheets (gray segments) by the interaction of their positively charged edges with anionic sodium alginate (red lines). b) Preparation of EISHs by dispersing clay nanosheets in 0.2 wt% sodium alginate. c) TEM images of the exfoliated clay nanosheets. d) Feasible injection of EISHs through a 25 gauge needle and the immediate reformation of a steady gel after injection.

Shear‐thinning is a term used in rheology to describe non‐Newtonian fluids which demonstrate viscous flow under shear stress and subsequent recovery upon removal of the stress.^28^ Recognizing this property, we hypothesized that shear‐thinning hydrogels could serve as a platform for endoscopic injection and cushion formation. We recognized Laponite, a layered nanosilicate with good biocompatibility and biodegradability, which is commonly utilized as a rheology modifier and additive to promote shear‐thinning and thixotropic behavior, as a material candidate for the synthesis of the gels.^29^, ^30^ (Laponite is a trademark of the company BYK Additives Ltd.) Alginate, an anionic polysaccharide extracted from seaweeds, is biocompatible and its aqueous solutions demonstrate non‐Newtonian fluid behavior with shear‐thinning properties.^31^ Here, we use alginate to exfoliate and disperse Laponite nanosheets by mutual repulsion resulting from a possible site‐specific wrapping of their positively charged edge parts^29^ with anionic alginate (**Figure** **1**a). Laponite was briefly exfoliated in 0.2 wt% sodium alginate aqueous solution and a transparent EISH was formed immediately after sonication (Figure 1b). Transmission electron microscopy (TEM) images show that Laponite nanosheets were dispersed homogenously (Figure 1c). The prepared EISHs could be easily injected through a 25 gauge needle and immediately reformed a solid gel after injection, as shown in Figure 1d.

### 2.2 Rheological Properties of EISHs

**Figure 2:**
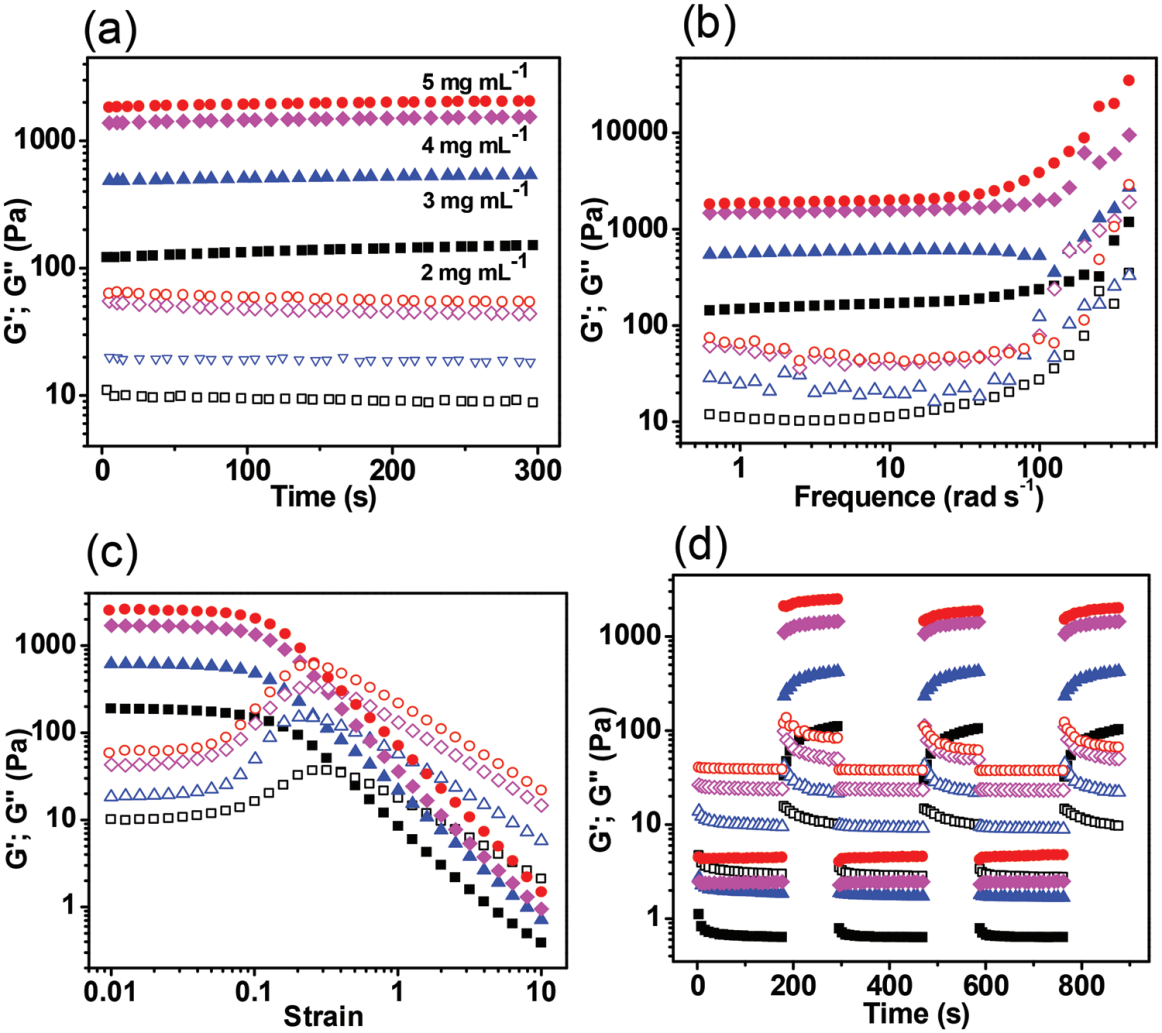
Rheological properties of EISHs. a) Oscillatory time sweeps of EISHs. Sweeps were performed at 0.5% strain and 6.3 rad s^−1^. b) Oscillatory frequency sweeps of EISHs. Sweeps were performed at 0.5% strain. c) Oscillatory strain sweeps of EISHs. Sweeps were performed at 6.3 rad s^−1^. d) Deformation and recovery of EISHs. Gels evolved over time from repeated cycles of 3 min low 0.5% strain and 2 min high 500% strain oscillations at 6.3 rad s^−1^. *G*′ (filled symbols) and *G*″ (empty symbols) represent storage and loss modulus, respectively.

We investigated the effect of Laponite concentration on the rheological behavior of EISHs. Oscillatory measurements indicate that both the storage modulus (*G*′) and loss modulus (*G*″) of EISHs increased with concentration. Time sweep experiments show that the *G*′ and *G*″ values of EISHs separately increased from ≈10 and ≈100 Pa to ≈70 and ≈1100 Pa with increasing Laponite concentration from 2 to 5 mg mL^−1^ (**Figure** **2**a). Frequency sweep measurements display that EISHs exhibited constant *G*′ values approximately 10–19 times higher than *G*″ values throughout the frequency sweep from 0.1 to 100 rad s^−1^, indicating the formation of stable hydrogels (Figure 2b). Strain‐dependent oscillatory rheology experiments were further performed to examine the linear viscoelastic range of EISHs. As shown in Figure 2c, the moduli of EISHs were independent of strain amplitude and showed linear viscoelastic behavior at low strain ranging from 0.01 to 0.1. Beyond their critical strains around 0.1, the *G*′ values of EISHs decreased rapidly with the increase of strain, suggesting the gels underwent gel–sol transition and behaved as liquids. The cross point of *G*′ and *G*″, representing the transition of the gel network to a liquid state (solution behavior: *G*′ < *G*″, solid behavior: *G*′ > *G*″), increased from ≈25 to ≈600 Pa with increasing Laponite concentration from 2 to 5 mg mL^−1^, respectively. These data demonstrate that the rheological behavior and shear‐thinning properties of EISHs can be conveniently tuned by adjusting Laponite concentrations.

Step‐strain measurements were performed to verify the reversible gel–sol transition of EISHs. The deformation and recovery of EISHs were conducted at repeated cycles of 3 min low‐magnitude strain of 0.5% and 2 min high‐magnitude strain of 500% oscillations at 6.3 rad s^−1^. After applying alternative low and high strains, we monitored the moduli of EISHs during the strain changes. As shown in Figure 2d, the gels underwent gel–sol transition and behaved as liquids upon increasing oscillatory strain from 0.5% to 500%. Inversely, EISHs rapidly underwent sol–gel transition and recovered back to their initial moduli immediately with lowering the strain from 500% to 0.5%. The gel–sol transition was reversible and all gels were capable of self‐healing to their original state without showing any signs that mechanical fidelity was compromised, irrespective of the number of times they were previously shear‐thinned. These data demonstrate the robust reversibility of the mechanical properties of EISHs.

### 2.3 In Vitro Evaluation of EISHs

**Figure 3:**
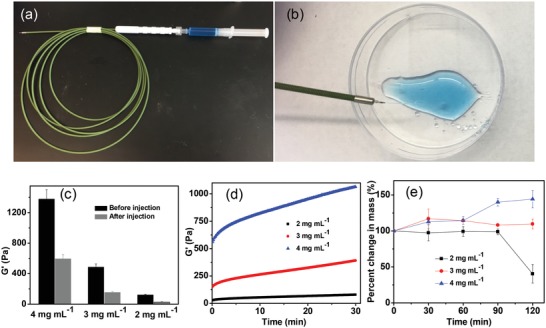
In vitro evaluation of EISHs. a) A photo of an endoscopic needle. b) Fluent injection of EISHs via the endoscopic needle. c) Storage modulus of EISHs before and after injection by a 25 gauge needle at an injection speed of 0.25 mL s^−1^. d) Gel restoration kinetics of EISHs subjected to shear force induced by syringe injection (*G*′ of 2 mg mL^−1^ at 0 min was 28 Pa and at 30 min was 79 Pa, *G*′ of 3 mg mL^−1^ at 0 min was 153 Pa and at 30 min was 391 Pa, and *G*′ of 4 mg mL^−1^ at 0 min was 561 Pa and at 30 min was 1064). e) Gel erosion kinetics of EISH cushions in saline at 37 °C. The error bars show standard deviation (*n* = 3).

We next studied the injection feasibility of EISHs by utilizing a standard 25 gauge endoscopic needle^32^ that is widely used for in vivo submucosal injection in endoscopic procedures (**Figure** **3**a). Representative formulations of EISHs with a Laponite concentration of 2 mg mL^−1^ could be injected as shown in Figure 3b. The storage modulus of EISHs with Laponite concentrations of 2, 3, and 4 mg mL^−1^ decreased from its initial *G*′ after passing through a 25 gauge needle with an injection speed of 0.25 mL s^−1^ to 23%, 31%, and 43%, respectively (Figure 3c). To elucidate the recovery capability of EISHs, oscillatory time sweep rheology measurements were performed immediately following the injection. As shown in Figure 3d, the modulus of EISHs with Laponite concentrations of 2, 3, and 4 mg mL^−1^ increased by 2.9, 2.6, and 1.9 times in 30 min, respectively. These results demonstrate the feasibility of injection of EISHs and their rapid conversion to a solid gel following injection.

We then evaluated the stability of EISHs by measuring their erosion kinetics in a physiological environment. A volume of 0.5 mL of EISHs was injected in saline and further incubated at 37 °C for predetermined time intervals. The volume of remaining gels at each time point was recorded to calculate the erosion kinetics of EISHs. As shown in Figure 3e, the mass of EISHs with Laponite concentration of 2 mg mL^−1^ remained constant within 1.5 h. While the mass of the gels decreased to 40% with further prolonging of incubation time to 2 h, which could be explained by the passive diffusion of both Laponite and alginate. However, EISHs with a higher Laponite concentration of 3 mg mL^−1^ maintained their mass up to 2 h. Interestingly, EISHs with a high concentration of 4 mg mL^−1^ swelled gradually and reached 1.4 times of their initial mass after 2 h incubation. We speculate that the dispersion of a high Laponite content of 4 mg mL^−1^ in alginate aqueous solution forms steady hydrogels that can promote their water absorption. These erosion profiles suggest the potential of EISHs to resist passive diffusion and achieve relative long‐term submucosal cushions.

### 2.4 Endoscopic Development of Submucosal Cushions

**Figure 4:**
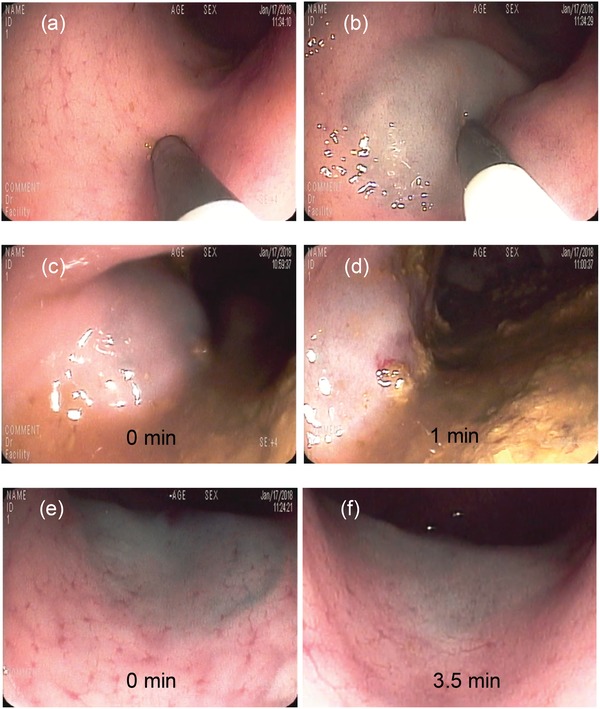
Endoscopic development of submucosal cushions. a,b) Pig colon before (a) and after (b) submucosal injection of 1.5 cc of 2 mg mL^−1^ EISH. c–f) Endoscopic images of the submucosal cushions developed by saline (c,d) and 2 mg mL^−1^ EISH (e,f) at different post‐injection time points, showing the significantly prolonged duration of the cushions developed by EISHs.

Having confirmed the feasible injection and rapid recovery as well as high stability of EISHs, we then tested their performances for cushion development in vivo. Yorkshire pigs weighing 40–80 kg were used as a large animal model and endoscopic injection was utilized to develop submucosal cushions in the colon. As displayed in **Figure** **4**a,b, a clear cushion was easily formed by submucosal injection of 1.5 cc of EISHs (2 mg mL^−1^) through an endoscopic needle. Four different injections were performed and each time a well‐formed cushion was observed. Additionally, endoscopic videography was used to observe the duration of cushions formed by EISHs. We found that the cushions created by normal saline flattened dramatically within 1 min (Figure 4c,d), while the cushions produced by EISHs remained almost unchanged for up to 3.5 min (Figure 4e,f), showing the prolonged duration of cushions developed by these gels.

**Figure 5:**
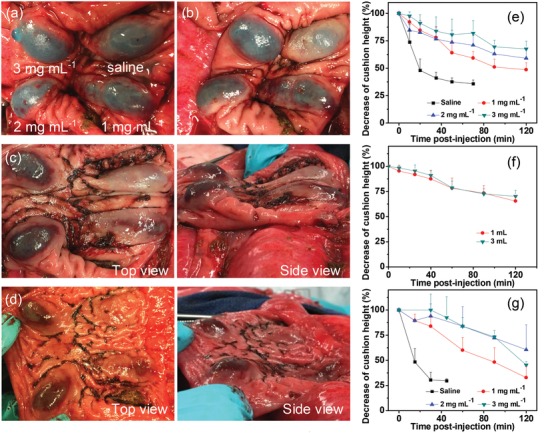
In vivo submucosal cushion duration. a–d) Photographs of submucosal cushions in pig colon post‐injection at: a) 0, b) 30, c) 60, and d) 120 min, respectively. The cushions were lifted by 2 cc of saline (top right), 1 mg mL^−1^ EISH (bottom right), 2 mg mL^−1^ EISH (bottom left), and 3 mg mL^−1^ EISH (top right), respectively. e) Duration of cushions lifted by 2 cc of EISHs with different concentrations in pig colon (*t* = 20 min, *p* < 0.05). No further changes observed in saline injection site. f) Relationship between time post‐injection and height of cushions developed by 2 mg mL^−1^ EISHs with different volumes (no significance). g) Duration of cushions lifted by 2 cc of EISHs with different concentrations in small intestine (*t* = 15 min, *p* < 0.05). 2 cc of saline solution was injected as a control. No further changes observed in saline injection site. The error bars show standard deviation (*n* = 3). Significance was assessed using Student's *t*‐test.

To accurately evaluate the duration of cushions, we measured their heights by a digital caliper through a midline laparotomy during a terminal procedure. 2 cc of EISHs with different Laponite concentrations were submucosally injected in pig colon and the heights of the resulted cushions were measured at predetermined time intervals. Normal saline was used as a control. As observed in **Figure** **5**a–d, with the increase of incubation time, the heights of cushions created by EISHs decreased at a far slower rate than that of saline. At 20 min incubation, the height of cushions developed by saline decreased dramatically to less than 50%, while the heights of all cushions created by EISHs with different concentrations from 1 to 3 mg mL^−1^ remained as high as 82% to 91% (Figure 5e). Even with incubation time prolonging to 2 h, the heights of cushions elevated by EISHs with a low Laponite concentration of 1 mg mL^−1^ remained around 50%. Notably, the cushions developed by EISHs with a Laponite concentration of 3 mg mL^−1^ maintained up to 68% of their initial heights after 2 h incubation, demonstrating the significantly prolonged durations. We further investigated the effect of injection volume on the duration of cushions. As illustrated in Figure 5f, with increasing injection volume from 1 to 3 mL, no obvious differences were observed between the heights of cushions produced by EISHs with a Laponite concentration of 2 mg mL^−1^. We also evaluated the durations of cushions elevated in pig small intestine. As expected, their durations were quite similar to those created in pig colon (Figure 5g), showing the capabilities of EISHs to develop durable submucosal cushions at different sites.

### 2.5 Local Toxicity of EISHs

**Figure 6:**
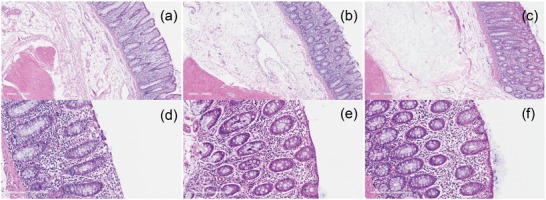
In vivo toxicity. a) H&E staining images of pig colon without any treatment, b) submucosally injected with saline, and c) 3 cc of 3 mg mL^−1^ EISH, respectively. The tissues were harvested at 2 h post‐injection. d) H&E staining images of pig colon without any treatment, e) incubated with saline, and f) 3 cc of 3 mg mL^−1^ EISH on the top of the mucus for 2 h, respectively.

To complete our evaluation of the advantages of EISHs as submucosal injection agents, we evaluated their local toxicity by histological analysis.^33^ Hematoxylin and eosin (H&E) staining was used to evaluate the toxicity of EISHs against the tissue of pig colon in vivo. 3 cc of EISHs with a Laponite concentration of 3 mg mL^−1^ was submucosally injected submucosally in the colon of a sedated pig and normal saline was used as a control. At 2 h post‐injection, the pig was euthanized, and the tissues were immediately harvested, fixed by formalin, and further embedded by paraffin. The resultant tissues were then sectioned and stained by H&E for microscopic imaging. As shown in **Figure** **6**a–c, no significant difference was observed between the tissues treated by EISHs and the control tissues injected with normal saline. Similar results were obtained by incubation of EISHs placed on the top of the mucus for 2 h (Figure 6d–f), supporting the low local toxicity of these gels using as cushion development agents. Further evaluation of local effects as well as long‐term impact on adjacent regions including draining lymph nodes will be required for successful future human translation.

## 3 Conclusions

In summary, we report the development and application of shear‐thinning hydrogels as safe and endoscopically injectable solutions capable of establishing durable submucosal cushions. We show that these shear‐thinning hydrogels can be rapidly prepared by dispersing commercially available Laponite into an aqueous solution of alginate and their rheological properties can be easily tuned by varying the concentrations of Laponite. We also show that these hydrogels can be injected through a standard endoscopic needle and further demonstrate their low toxicity as well as the significantly enhanced durations of cushions elevated by these gels. In sum, the hydrogel materials developed herein present 1) commercially available and inexpensive resources; 2) tunable shear‐thinning properties and endoscopically injectable capability; 3) good biocompatibility and significantly improved stability for the development of durable submucosal cushions. All these features make EISHs a promising set of hydrogel materials for broad application in mucosal resection techniques and potentially luminal constriction, drug delivery, and tissue engineering.

## 4 Experimental Section

*Materials*: Sodium alginate, Laponite, Indigo carmine, methylene blue, and other chemical reagents were purchased from Sigma and used as received unless otherwise noted. Nanopure water (18 MΩ cm) was acquired by means of a Milli‐Q water filtration system, Millipore (St. Charles).

*TEM Measurements*: TEM experiments were carried out on a JEOL 2100 FEG instrument at an acceleration voltage of 200 kV. The TEM sample was prepared by dropping the exfoliated Laponite solutions onto a 300‐mEISH carbon‐coated copper grid. Samples were blotted away after 30 min incubation at the room temperature and then washed twice with distilled water and air dried prior to imaging.

*Preparation of EISHs*: 0.2% sodium alginate aqueous solution was prepared as stock solution. Laponite was added into the stock solution with various concentrations and then sonicated for ≈2–5 min to obtain EISHs. EISHs with Laponite concentrations of 2, 3, 4, and 5 mg mL^−1^ were prepared accordingly and used directly for further measurements.

*Measurements of the Rheological Properties of EISHs*: Dynamic oscillatory time, frequency, and strain sweeps were performed using an AR2000 stress‐controlled rheometer (TA Instruments, New Castle, DE) with 25 mm steel plate geometry at a 27 mm gap distance. Laponite was dispersed in 0.2 wt% alginate solution by sonication to form EISHs with specified compositions and the gels were applied between the two plates of the rheometer. The top plate was lowered to a 27 mm gap distance and excess gel was scraped off. Care was taken to achieve a homogenous distribution of gel within the top and bottom plates of the rheometer. Dynamic oscillatory time sweeps were collected at angular frequencies of 6.3 rad s^−1^ and 0.5% strain. An initial strain amplitude sweep was performed at 25 °C at different frequencies to determine the linear viscoelastic range for the gels. Rheological properties were examined by frequency sweep experiments at fixed strain amplitude of 0.5%. Experiments were repeated on three to four samples and representative data were presented. For shear recovery experiments at 6.3 rad s^−1^, shear thinning was induced via application of 500% strain for 2 min. The strain was released to 0.5% for 3 min to allow the gel to recover.

*Erosion Studies of EISHs*: The erosion kinetics of the EISHs was measured in a physiological environment. A volume of 0.5 mL of EISHs was injected in saline and further incubated at 37 °C for 30, 60, 90, and 120 min, respectively. The volume of remaining gels at each time point was recorded to calculate the erosion kinetics of EISHs.

*Ex Vivo Cushion Development in Pig Colon*: Ex vivo cushion development was performed by injection of 0.5 cc EISHs (2 mg mL^−1^) into the pig colon. The colon tissue was isolated from freshly procured intact gastrointestinal tracts from pigs from selected local slaughter houses. The top view and the side view of the developed cushions were shown in Figure S1 in the Supporting Information.

*In Vivo Cushion Development in a Pig Model*: All pig experiments were approved by the Committee on Animal Care at the Massachusetts Institute of Technology. Female Yorkshire pigs (40–80 kg) were obtained from Tufts University and housed under conventional conditions. Animals were randomly selected for the experiments. The animals were placed on a liquid diet for 24 h prior to the experiment with the morning feed held on the day of the experiment. At the time of the experiment, the pigs were anesthetized with intramuscular administration of Telazol (tiletamine/zolazepam, 5 mg kg^−1^), xylazine (2 mg kg^−1^), and atropine (0.04 mg kg^−1^). An endoscope (Pentax, US endoscopy) was inserted into the distal colon and a Carr–Locke needle was inserted through the channel of the endoscope into the colon. Subsequently, 1.5 mL of saline and hydrogel were separately injected into the submucosal space, repeated three times. Videos were recorded to monitor the decrease of the size of the cushions lift. All animals were recovered from anesthesia.

*Measurements of In Vivo Cushion Duration*: All procedures were conducted in accordance with protocols approved by the Massachusetts Institute of Technology Committee on Animal Care. Female Yorkshire swine, approximately 40–80 kg in body weight were anesthetized with intramuscular administration of Telazol (tiletamine/zolazepam, 5 mg kg^−1^), xylazine (2 mg kg^−1^), and atropine (0.04 mg kg^−1^). Animals were intubated and maintained on 2–3% isoflurane in oxygen. As part of a terminal or nonsurvival procedure, a midline laparotomy was performed and the proximal jejunum or distal colon was accessed and stabilized with gauze. A longitudinal incision was made to access the luminal side and 2 cc normal saline solution and 1 mg mL^−1^ EISH, 2 mg mL^−1^ EISH, and 3 mg mL^−1^ EISH were injected into the submucosal space to form the cushions. The length, width, and the height of the cushions were measured at 0, 30, 60, and 120 min after the injection. 1, 2, and 3 mL 2 mg mL^−1^ EISHs were also injected to investigate the cushion properties. Animal were euthanized prior to anesthetic recovery with intravenous administration of 120 mg kg^−1^ of sodium pentobarbital.

*H&E Staining*: The toxicity of EISHs was evaluated during an in vivo terminal experiment. All procedures were conducted in accordance with protocols approved by the Massachusetts Institute of Technology Committee on Animal Care. Pigs were intubated and maintained on 2–3% isoflurane in oxygen. A midline laparotomy was performed and the proximal jejunum was accessed and stabilized with gauze. 3 cc normal saline solution and 3 mg mL^−1^ EISH were submucosally injected to the pig colon to form the cushions. Meantime, multiple 4–5 cm incisions were made along the antimesenteric side of the colon. 3 cc normal saline solution and 3 mg mL^−1^ EISH were incubated on the top of the mucus using wells secured with carbopol and covered with an adhesive membrane. The pigs were euthanized with sodium pentobarbital (120 mg kg^−1^) intravenously prior to tissue collection. Tissues were harvested and placed into formalin (4%). After tissues were fixed in formalin, they were paraffin embedded, sectioned, and stained with H&E for analysis.

## Conflict of Interest

Y.P., J.L., R.L. and G.T. are co‐inventors on a provisional patent application encompassing the technology described in this manuscript.

## Supporting information

Supplementary

Supplementary

Supplementary

Supplementary

Supplementary
