# Supplementary material for: Endoscopically Injectable Shear‐Thinning Hydrogels Facilitating Polyp Removal
Source: Adv Sci (Weinh). 2019 Jul 30;6(19):1901041. doi: 10.1002/advs.201901041 (PMC6774062; doi:10.1002/advs.201901041)
Supplement: Supplementary file 1 — Supplementary [file ADVS-6-1901041-s002.pdf]

Copyright WILEY-VCH Verlag GmbH & Co. KGaA, 69469 Weinheim, Germany, 2019.

ADVANCED SCIENCE
Open Access

Supporting Information

for *Adv. Sci.,* DOI: 10.1002/advs.201901041

Endoscopically Injectable Shear-Thinning Hydrogels Facilitating Polyp Removal

*Yan Pang, Jinyao Liu, Zaina L. Moussa, Joy E. Collins, Shane McDonnell, Alison M. Hayward, Kunal Jajoo, Robert Langer, and Giovanni Traverso**

Supporting Information

Endoscopically-injectable shear-thinning hydrogels facilitating polyp removal

Yan Pang, Jinyao Liu', Zaina L Moussa, Joy E Collins, Shane McDonnell, Alison M Hayward, Kunal Jajoo, Robert Langer, Giovanni Traverso[*]

Dr. Y. Pang,

Department of Ophthalmology, Ninth People's Hospital, Shanghai Key Laboratory of Orbital Diseases and Ocular Oncology, Shanghai Jiao Tong University School of Medicine, Shanghai 200011, China

Department of Chemical Engineering and Koch Institute for Integrative Cancer Research, Massachusetts Institute of Technology, Cambridge, Massachusetts 02139, USA.

Prof. J. Y. Liu,

Institute of Molecular Medicine, State Key Laboratory of Oncogenes and Related Genes, Shanghai Institute of Cancer, Renji Hospital, Shanghai Jiao Tong University School of Medicine, Shanghai 200127, China

Department of Chemical Engineering and Koch Institute for Integrative Cancer Research, Massachusetts Institute of Technology, Cambridge, Massachusetts 02139, USA.

Z. L. Moussa, J. E. Collins, S. McDonnell

Department of Chemical Engineering and Koch Institute for Integrative Cancer Research, Massachusetts Institute of Technology, Cambridge, Massachusetts 02139, USA.

A. M. Hayward

Department of Chemical Engineering and Koch Institute for Integrative Cancer Research, Division of Comparative Medicine, Massachusetts Institute of Technology, Cambridge, Massachusetts 02139, USA.

K. Jajoo

Division of Gastroenterology, Brigham and Women's Hospital, Harvard Medical School, Boston, Massachusetts 02115, USA.

Prof. R. Langer

Department of Chemical Engineering and Koch Institute for Integrative Cancer Research, Harvard–MIT Division of Health Sciences and Technology, Massachusetts Institute of Technology, Cambridge, Massachusetts 02139, USA.

Prof. G. Traverso

Department of Chemical Engineering and Koch Institute for Integrative Cancer Research, Department of Mechanical Engineering, Massachusetts Institute of Technology, Cambridge, Massachusetts 02139, USA.

Division of Gastroenterology, Brigham and Women's Hospital, Harvard Medical School, Boston, Massachusetts 02115, USA.

*e-mail: ctraverso@partners.org, cgt20@mit.edu

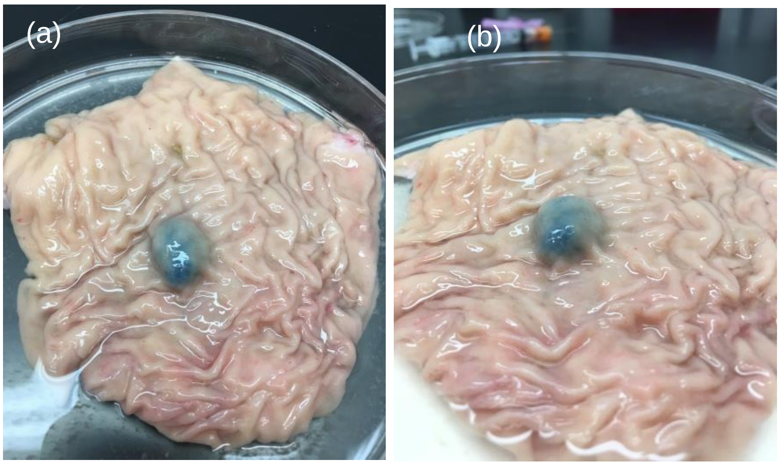

Figure S1. Ex vivo cushion development in pig colon by injection of EISHs. (a) top view; (b) side view.

Video 1. Injection of EISHs via a 25-gauge needle and the recovery of a solid gel

Video 2. Injection of EISHs via a standard endoscopic needle

Video 3. Formation of a stable solid gel in PBS after injection

Video 4. In vivo endoscopic injection of EISHs using a pig model
